# More than 2500 years of oil exposure shape sediment microbiomes with the potential for syntrophic degradation of hydrocarbons linked to methanogenesis

**DOI:** 10.1186/s40168-017-0337-8

**Published:** 2017-09-11

**Authors:** Antonios Michas, Gisle Vestergaard, Kathleen Trautwein, Pavlos Avramidis, Dimitris G. Hatzinikolaou, Constantinos E. Vorgias, Heinz Wilkes, Ralf Rabus, Michael Schloter, Anne Schöler

**Affiliations:** 1 Research Unit Comparative Microbiome Analysis (COMI), Helmholtz Zentrum München, Ingolstaedter Landstraße 1, D-85764 Neuherberg, Germany; 2 General and Molecular Microbiology, Institute for Chemistry and Biology of the Marine Environment (ICBM), Carl von Ossietzky University Oldenburg, Carl-von-Ossietzky-Straße 9-11, 26111 Oldenburg, Germany; 3 Department of Geology, University of Patras, Panepistimioupoli Patron, 26504 Rio-Patras, Greece; 4 Department of Biology, National and Kapodistrian University of Athens, Zografou University Campus, 15784 Athens, Greece; 5 Organic Geochemistry, Institute for Chemistry and Biology of the Marine Environment (ICBM), Carl von Ossietzky University Oldenburg, Carl-von-Ossietzky-Straße 9-11, 26129 Oldenburg, Germany

**Keywords:** Keri Lake, Asphalt oil, Long-term exposure, Anaerobic degradation of hydrocarbons, Methanogenesis, Syntrophic interactions, Metagenomic shotgun sequencing

## Abstract

**Background:**

Natural oil seeps offer the opportunity to study the adaptation of ecosystems and the associated microbiota to long-term oil exposure. In the current study, we investigated a land-to-sea transition ecosystem called “Keri Lake” in Zakynthos Island, Greece. This ecosystem is unique due to asphalt oil springs found at several sites, a phenomenon already reported 2500 years ago. Sediment microbiomes at Keri Lake were studied, and their structure and functional potential were compared to other ecosystems with oil exposure histories of various time periods.

**Results:**

Replicate sediment cores (up to 3-m depth) were retrieved from one site exposed to oil as well as a non-exposed control site. Samples from three different depths were subjected to chemical analysis and metagenomic shotgun sequencing. At the oil-exposed site, we observed high amounts of asphalt oil compounds and a depletion of sulfate compared to the non-exposed control site. The numbers of reads assigned to genes involved in the anaerobic degradation of hydrocarbons were similar between the two sites. The numbers of denitrifiers and sulfate reducers were clearly lower in the samples from the oil-exposed site, while a higher abundance of methanogens was detected compared to the non-exposed site. Higher abundances of the genes of methanogenesis were also observed in the metagenomes from other ecosystems with a long history of oil exposure, compared to short-term exposed environments.

**Conclusions:**

The analysis of Keri Lake metagenomes revealed that microbiomes in the oil-exposed sediment have a higher potential for methanogenesis over denitrification/sulfate reduction, compared to those in the non-exposed site. Comparison with metagenomes from various oil-impacted environments suggests that syntrophic interactions of hydrocarbon degraders with methanogens are favored in the ecosystems with a long-term presence of oil.

**Electronic supplementary material:**

The online version of this article (10.1186/s40168-017-0337-8) contains supplementary material, which is available to authorized users.

## Background

Hydrocarbons are widespread in nature and have either geochemical or biological origin [1]. Most of them are energy-rich but also relatively stable due to the inertness of their chemical bonds [2]. Despite this, many microorganisms are able to degrade hydrocarbons under oxic [3–5] or anoxic conditions [6]. Under anoxic conditions, microbes respire electron acceptors other than molecular oxygen (O_2_), including nitrate, iron, and sulfate, or thrive in syntrophic methanogenic consortia. In the latter case, hydrocarbons are degraded by fermenting bacteria to intermediate products (e.g., H_2_, acetate), which are subsequently converted to CH_4_ by methanogens [7].

Compared to the aerobic degradation of hydrocarbons, our understanding is less comprehensive about the enzymatic reactions and the key players of the anaerobic pathways. One of the best studied mechanisms to activate chemically stable hydrocarbons under anoxic conditions is catalyzed by alkyl-/arylalkylsuccinate synthases. This involves the addition of methyl and methylene groups of *n*-alkanes or aromatic hydrocarbons to fumarate. The respective genes have been detected in denitrifying [8–12], iron-reducing [13], sulfate-reducing [14–16], and syntrophic [17] prokaryotes. Alternative activation mechanisms involve O_2_-independent hydroxylation [11, 18] or putative carboxylation of aromatic hydrocarbons [19, 20]. Many anaerobic peripheral degradation pathways converge at central intermediates, like benzoyl-CoA in the case of aromatic hydrocarbons, and are ultimately channeled into the central metabolism via acetyl-CoA [21, 22] for terminal oxidation to CO_2_.

The dynamics of aerobic and anaerobic hydrocarbon degraders have been studied mainly in the context of short-term oil contamination of marine open waters, sediments or coastal environments in the aftermath of large-scale accidents, including the Nakhodka [23, 24], the Prestige [25], and the Deepwater Horizon [26–32] oil spills. In contrast, our knowledge about the effects of long-term exposure to oil on the microbiomes of soils, sediments, and waterbodies is limited to studies on offshore submarine oil seeps [33], subsurface oil reservoirs [34, 35], or oil-contaminated environments [36–38]. These studies have investigated the microbial diversity and identified sulfate reducers and/or methanogens to be abundant; however, a connection of redox processes to the catabolism of available carbon sources is missing.

Therefore, the aim of this study was to identify the major redox processes in long-term oil-exposed sediments and link them to the transformation of hydrocarbons present in the crude oil. Thus, we investigated a fen ecosystem in a land-to-sea transition zone called “Keri Lake”, which is located in the southwestern part of Zakynthos Island, Western Greece [see Additional file 1: Figure S1]. This ecosystem is unique due to several sites with asphalt oil springs, mentioned by the ancient Greek historian Herodotus (Book IV, Chapter 195) already 2500 years ago. The oil source rock is located below the neighboring Marathonisi Island. Oil migrates through rock fractures towards the soil surface and escapes in the area of Keri Lake and near Marathonisi [39]. During this migration, microbial degradation under the prevailing anoxic conditions is expected to significantly modify the composition of oil and deplete inorganic electron acceptors (nitrate and sulfate). We hypothesize that the anaerobic catabolic potential of the sediment microbiomes is influenced by the presence of asphalt oil and that syntrophic interactions with methanogens are favored, due to the lack of electron acceptors. To test our hypothesis we used a metagenomic sequencing approach, based on DNA extracted from different depths of an oil-exposed site as well as a non-exposed control site at Keri Lake, and focused on genes coding for enzymes involved in the anaerobic degradation of hydrocarbons and the respiratory pathways of interest. To see if our findings can also be observed in other ecosystems, we further extended our analysis to include other metagenomic datasets generated from samples with long histories of oil exposure.

## Methods

### Study site and sampling campaign

Keri Lake spans an area of 30,000 m^2^ and is lying 1 m above the sea level. It is separated from the open sea by a partially artificial low relief sand barrier, which limits the exchange of (sub)surface waters with the sea. The ancient lake environment has turned into a coastal middle-brackish to freshwater fen, overgrown mainly with reeds. Recent geological studies revealed the accumulation of a ca. 5-m-thick peat bed below the fen environment [40, 41].

Sampling was conducted with permissions from the Greek National Focal Point to the Convention on Biological Diversity/Ministry of Environment, Energy and Climate Change, as well as from the National Marine Park of Zakynthos. Duplicate sediment cores up to 3-m depth were collected in October 2013 from two sites in Keri Lake: (i) NE, non-exposed to oil (by visual and olfactory inspection; 37° 41.112 N, 20° 49.769 E) and (ii) HE, highly exposed to oil (37° 41.169 N, 20° 49.900 E) [see Additional file 1: Figure S1C]. The distance between duplicate cores ranged from 0.5 to 1 m. The cores were obtained with stainless steel tubing (diameter 5–10 cm) using a vibrating corer (Eijkelkamp Soil & Water, Giesbeek, The Netherlands). After carefully removing the sediment that was in contact with the tubing, samples were collected on site from three different depth zones: 40–60 cm, 130–180 cm, and 210–250 cm. Top soil was excluded from the analysis, due to past human intervention. For the analysis of the hydrocarbons, 0.5–4.2 g of sediment was stored at 4 °C until further processing. For the chemical analyses of nitrate and sulfate, sediment samples (1.5–5.0 g) were incubated for 60 min with 10 ml of 1 M KCl, from which 8 ml were subsequently filtered with sterile Millex–GP filter units (0.22 μm; Merck Millipore, Billerica, Massachusetts, USA) and stored at 4 °C. For the analysis of the microbial communities, approximately 3 g of sediment was mixed with 4.5 ml of sodium phosphate buffer (112.9 mM Na_2_HPO_4_, 7.1 mM NaH_2_PO_4_, pH 8.0) and 1.5 ml of TNS solution (10% *w*/*v* sodium dodecyl sulfate, 0.5 M Tris-HCl at pH 8.0, 0.1 M NaCl) in bead beating vials and was immediately frozen in liquid nitrogen and stored at −80 °C until further analysis. Last, a few grams of sediment (0.1–2.5 g) from each depth were incubated at 60 °C until constant weight, in order to determine the dry weight of the samples. Samples from the same depths of the duplicate cores at each site were used as biological replicates.

### Nitrate and sulfate analysis

Nitrate and nitrite concentrations were measured using a San++ Continuous Flow Analyzer, with a detection limit of 60 and 20 μg l^−1^, respectively (Skalar Analytical B.V., Breda, The Netherlands).

Sulfate concentrations were determined by ion chromatography using an ICS–1100 (ThermoFisher Scientific Inc., Waltham, Massachusetts, USA), equipped with an IonPac AG-18 pre-column (4 × 50 mm, 13 μm bead size; ThermoFisher Scientific Inc.) and an AS-18 anion-exchange separation column (4 × 250 mm, 7.5 μm bead size; ThermoFisher Scientific Inc.). Sulfate was eluted at 23 min with 10 mM KOH as the eluent delivered at a flow rate of 1.5 ml min^−1^; the detection limit was 5 μg l^−1^.

### Hydrocarbon analysis

The respective sediment samples were extracted for 24 h in a Soxhlet apparatus using dichloromethane:methanol (99:1, *v*/*v*; Sigma-Aldrich Inc., St. Louis, Missouri, USA). After complete removal of the solvent by evaporation, the extractable organic matter (EOM) yields were determined gravimetrically. The residues were taken up in a small amount of dichloromethane for asphaltene precipitation [42], after which the maltene fractions were separated into saturated hydrocarbon, aromatic hydrocarbon, and non-hydrocarbon fractions using medium-pressure liquid chromatography [43]. The saturated hydrocarbon fractions were analyzed by gas chromatography with flame ionization detection, as described by Hosseini et al. [44].

### Nucleic acid extraction

Total DNA from the buffered sediment samples was extracted using the protocol of Lueders et al. [45], with the following modifications: 1 ml of phenol:chloroform:isoamylalcohol (P/C/I; 25:24:1, *v*/*v*/*v*; Carl Roth GmbH + Co, Karlsruhe, Germany) was added to the frozen/thawed samples, followed by incubation at 65 °C for 10 min prior to two consecutive steps of cell destruction by bead beating (6500 rpm, 60 s). After centrifugation (4000×*g*, 5 min), DNA from the aqueous supernatants was purified with equal volumes of P/C/I (25:24:1, *v*/*v*/*v*; Carl Roth GmbH + Co) and subsequently chloroform:isoamylalcohol (C/I; 24:1, *v*/*v*; Carl Roth GmbH + Co). Purified DNA was precipitated with a mixture of isopropanol (0.8 × sample volume; AppliChem GmbH, Darmstadt, Germany) and 3 M sodium acetate (0.1 × sample volume) at −20 °C overnight, followed by centrifugation (4000×*g*, 2 h) and washing with 3 ml ice-cold ethanol (70%, *v*/*v*). Pelleted DNA was resuspended in TE buffer (1 ×, pH 7.5; AppliChem GmbH) and then purified using the OneStep PCR Inhibitor Removal Kit (Zymo Research Corp., Irvine, California, USA) according to the manufacturer’s protocol. One non-template sample (molecular-grade distilled water) was included as a negative control during the whole workflow.

### DNA sequencing, quality control of sequenced reads and phylogenetic analysis

1.5 ml of each DNA sample was further purified using the Genomic DNA from Soil Kit (Macherey-Nagel GmbH&Co. KG, Düren, Germany), excluding the homogenization and lysis steps of the manufacturer’s protocol. Purified DNA was sheared in an E220 Focused-ultrasonicator (Covaris Inc., Woburn, Massachusetts, USA) with the following parameters: peak incident power 175 W, duty factor 10%, 200 cycles per burst, and treatment time 100 s. Sheared DNA was purified using Agencourt AMPure XP beads (1.8 × sample volume; Beckman Coulter Inc., Brea, California, USA) and was concentrated to 55 μl; the final DNA amount of the samples ranged from 0.3 to 1.0 μg. Metagenomic libraries were prepared using the NEBNext Ultra DNA Library Prep kit for Illumina (New England BioLabs LtD., Hitchin, UK). Before adaptor ligation, the provided adaptor was diluted with molecular-grade water for samples with low starting material (1:2 for samples with 0.5–0.7 μg or 1:3 for samples with 0.3–0.5 μg and the non-template control). Finally, the adaptor-ligated DNA was purified with size selection and amplified during nine PCR cycles. Metagenomic libraries were sequenced on a MiSeq instrument (Illumina Inc., San Diego, California, USA) using the MiSeq Reagent Kit v3 for 600 cycles. One replicate of each sampling depth and site was sequenced with a depth of > 2 million reads, the other replicate with a depth of > 1 million reads and used to compare with the deep-sequenced replicate, check for heterogeneity of the sites and test for uniformity.

Raw datasets were processed following a bioinformatics pipeline adjusted to Illumina sequencing [46], with the following modifications. First, the Illumina adapters were identified and removed from the metagenomic reads with AdapterRemoval v2.1.0 [47]. Using the same program, paired reads were additionally merged, trimmed based on their quality (> 15), and filtered based on their length (> 100 bp). After removing phiX contaminants with DeconSeq [48], all quality-controlled reads were aligned to sequences of the NCBI non-redundant protein database (October 2015) using DIAMOND v0.5.2.32 [49]. Output data were analyzed with the MEGAN5 analyzer v5.7.1 [50], using the following settings: Min Score = 50, Top Percent = 10, Min Support = 1, Min-Complexity Filter = 0. All steps were parallelized using GNU Parallel [51]. Data visualization was performed with the statistical program R v3.2.2 [52]. Absolute counts were normalized by subsampling all datasets to an equal sequencing depth using the Rarefy command of the GUniFrac package [53]. Non-metric multidimensional scaling (NMDS) analysis was performed using the vegan package [54] and the ternary plots using the ternaryplot() function in the vcd package [55].

### Reconstruction of metabolic pathways

Rarefaction analysis for the functional potential of the 10 most abundant phyla was performed by extracting the reads annotated to these phyla and subsequently aligning them to sequences of the KEGG database (June 2011). To test our hypothesis though, we analyzed only the genes coding for enzymes involved in the complete anaerobic degradation of hydrocarbons to CO_2_ and the linked respiratory pathways. Thus, metagenomic reads were annotated to the genes coding for enzymes catalyzing the anaerobic degradation of hydrocarbons, the TCA cycle, the Wood-Ljungdahl pathway, denitrification, sulfate reduction, and methanogenesis (68 genes in total). For each gene, an individual database was compiled, which consisted of representative protein sequences of all enzyme subunits [see Additional file 2: Table S1]. Protein sequences were obtained from the UniProtKB database [56]; sequences obtained by whole genome sequencing were preferred. The matched metagenomic reads were filtered by identity (≥ 50%), alignment length (≥ 50 bp), and bit score (≥ 50) and subjected to taxonomic affiliation, normalization, and visualization, using DIAMOND, MEGAN5, and R as described above.

### Comparative analysis of metagenomes

The unassembled reads from the metagenomes of the samples from Keri Lake were additionally uploaded and analyzed on the MG-RAST server [57], to allow a direct comparison with other publically available datasets. Paired reads were merged using the default options. Artificial replicates and low-quality sequences were removed with lowest phred score 20 and the remaining sequences were trimmed when five consecutive bases below the phred score cut-off occurred. Metagenomic sequences are available under the following MG-RAST IDs: 4663010.3, 4663012.3, 4663014.3, 4663018.3, 4663020.1, and 4663021.3. Additional metagenomes on MG-RAST were chosen, representing environments with oil contamination histories of various time periods [see Additional file 3: Table S2]. Short reads (< 100 bp) were removed from all metagenomes and the remaining sequences were aligned to the NCBI non-redundant and the manually compiled databases using DIAMOND and MEGAN5, as described above. The results were normalized using R by calculating relative abundances; rarefying was avoided due to the low number of sequenced reads of some publicly available metagenomes.

## Results

### Electron acceptors and hydrocarbons

At depth 40–60 cm, nitrate concentrations ranged from 0.03 to 0.08 mg g^−1^ dry sediment and sulfate concentrations from 0.5 to 1.1 mg g^−1^ dry sediment at the NE site [see Additional file 4: Figure S2A and Additional file 5: Table S3]. In contrast, higher nitrate concentrations were observed (0.33–0.35 mg g^−1^ dry sediment) at the HE site while the concentrations of sulfate were much lower (~ 0.006 mg g^−1^ dry sediment). At the depths of 130–180 cm and 210–250 cm, the concentrations of both electron acceptors were strongly reduced compared to those of the upper sediment layer at both sites. Nitrite was below the detection limit in all samples.

Extractable organic matter (EOM) yield was clearly higher at the HE (45.2–515.7 mg g^−1^ sediment) compared to that at the NE site (1.1–23.7 mg g^−1^ sediment) [see Additional file 5: Table S3]. The EOM yield increased with depth at the NE site, while no clear depth-related trend was observed for the HE site. Gas chromatographic analyses of the 130–180-cm depth samples revealed abundant *n*-alkanes (C_15_–C_35_) with a predominance of odd over even numbers of carbon atoms at the NE site, particularly in the range of C_25_–C_35_ [see Additional file 4: Figure S2B, upper panel]. At the HE site, a prominent unresolved complex mixture of saturated hydrocarbons was superimposed by hydrocarbon biomarkers with a hopane carbon skeleton [see Additional file 4: Figure S2B, lower panel].

### Taxonomic profiling

**Fig. 1:**
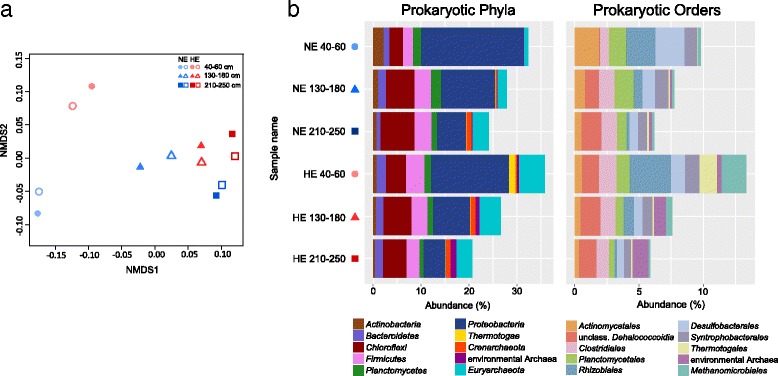
Taxonomic profiles of the study sites. **a** Non-metric multidimensional scaling (stress value = 0.03) of replicate samples from both sampling sites, based on the total taxonomic profile. Low-sequenced samples are depicted with no fill color. **b** Taxonomic annotation of the metagenomic reads from the deep-sequenced samples, showing the relative abundance of prokaryotic phyla and orders. The 10 most abundant taxa are included in each graph; the remaining taxa have less than 1% and 1.1% relative abundance at the phylum and order level, respectively

The number of sequenced reads ranged from 2 to 9 million for the deep-sequenced replicate samples and 0.6 to 2 million for the low-sequenced replicate samples [see Additional file 5: Table S3]. Only 568 reads were obtained for the non-template control, indicating very low contamination during DNA extraction and subsequent library preparation. Rarefaction curves of the datasets approached a saturation plateau at both the phylum and order level, especially for the deep-sequenced samples [see Additional file 6: Figure S3]. After normalization of all samples to ~ 0.66 million reads, biological replicates clustered closely in the NMDS plot (Fig. 1a). Therefore, all further analyses focused on the deep-sequenced metagenomes, normalized to ~ 2.12 million reads. The low-sequenced metagenomes were also analyzed but considered only to confirm the observations of the in-depth analyses.

Approximately 50–60% of the total reads could be assigned at the superkingdom level. Most reads were affiliated to prokaryotes, while less than 0.5% to eukaryotes and viruses. Figure 1b depicts the relative abundance of the most abundant phyla and orders. *Beta-*, *Gamma-*, and *Deltaproteobacteria* (e.g., *Desulfobacterales*) were ~ 2 times less abundant at the HE site, while *Clostridiales*, *Rhizobiales* (particularly the genus *Methyloceanibacter*), and *Thermotogales* (*Mesotoga* genus) showed a 2–10 times higher abundance, especially at 40–60-cm depth. Interestingly, the numbers of archaeal reads were two to six times higher at the HE compared to those at the NE site. Most reads were assigned to the phylum *Euryarchaeota* (mainly *Methanomicrobiales*), which includes methanogens. At the NE site, most archaeal orders were restricted to 210–250-cm depth, while at the HE site archaeal abundances were high irrespective of the depth [see Additional file 7: Figure S4]. The same patterns were observed for the metagenomes sequenced with low depth. When only 16S rRNA gene sequences were subsampled and analyzed, many reads assigned to *Thaumarchaeota* emerged in all metagenomes, but this did not affect the abovementioned differences between the samples (data not shown).

### Respiration processes and pathways of anaerobic degradation of hydrocarbons to CO_2_

Rarefaction curves on the functional potential reached a saturation plateau [see Additional file 8: Figure S5] indicating that the entire set of functions were covered for the main phyla and our sequencing depth was sufficient to detect all the genes of interest in this study.

**Fig. 2:**
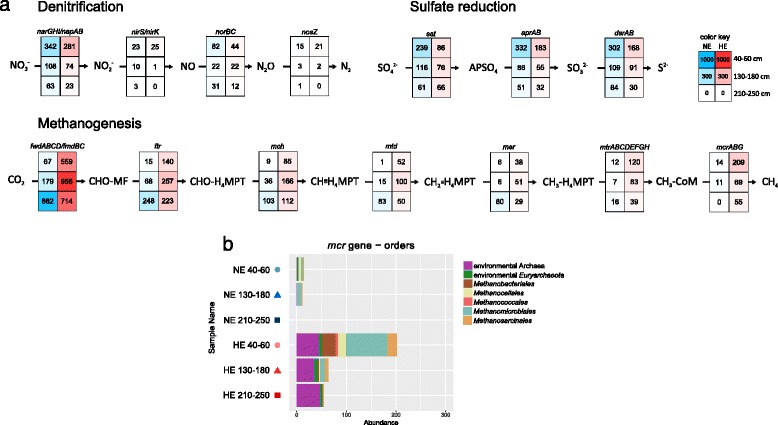
Anaerobic respiration processes. **a** Reconstruction of selected respiratory pathways: denitrification, sulfate reduction, and methanogenesis. The normalized absolute abundances of the genes catalyzing each step are given in the respective cells. **b** Taxonomic annotation of the metagenomic reads associated to the *mcr* gene. The seven most abundant orders are shown; each of the remaining orders represented less than four reads in the samples. Enzyme names: *narGHI/napAB*, nitrate reductase; *nirS/nirK*, nitrite reductase; *norBC*, nitric oxide reductase; *nosZ*, nitrous oxide reductase; *sat*, sulfate adenylyltransferase; *aprAB*, adenylylsulfate reductase; *dsrAB*, dissimilatory sulfite reductase; *fwdABCD/fmdBC*, formylmethanofuran dehydrogenase; *ftr*, formylmethanofuran-tetrahydromethanopterin *N*-formyltransferase; *mch*, methenyltetrahydromethanopterin cyclohydrolase; *mtd*, methylenetetrahydromethanopterin dehydrogenase; *mer*, 5,10-methylenetetrahydromethanopterin reductase; *mtrABCDEFGH*, tetrahydromethanopterin S-methyltransferase; *mcrABG*, methyl-coenzyme M reductase

At the NE site, high abundances of genes related to denitrification and sulfate reduction were only observed in the upper horizon, while those for methanogenesis in most cases showed highest numbers in the lower sediment layer (Fig. 2a). In contrast at all depths at the HE site, reads associated to genes coding for enzymes driving denitrification and sulfate reduction had generally 1.5–2 times lower abundances compared to those at the NE site. The genes of methanogenesis displayed higher abundances, particularly > 8 times higher for most genes at 40–60-cm depth, confirming the taxonomic data. Most reads associated to the methyl-coenzyme M reductase gene (*mcr*) were assigned to *Methanomicrobiales*, but *mcr* sequences from other methanogens were detected as well (Fig. 2b).

**Fig. 3:**
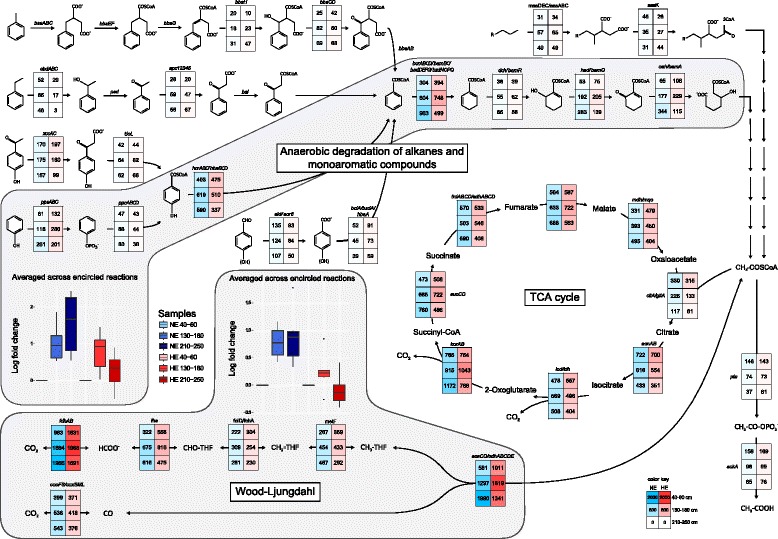
Reconstruction of the complete degradation of *n*-alkanes and monoaromatic compounds to CO_2_. The normalized absolute abundances of the genes for each step are given in the respective cells. The selection of the genes for the anaerobic degradation of aromatic compounds was based on the proteogenomics-based reconstruction of their catabolism in the denitrifying *Aromatoleum aromaticum* EbN1 [78] and the sulfate-reducing *Desulfobacula toluolica* Tol2 [15]. Only genes with abundances higher than 30 reads in at least one sample are presented. The boxplots depict the log fold changes of the abundances of all genes coding for the enzymes of the anaerobic degradation of phenolic compounds and the Wood-Ljungdahl pathway, respectively. Each sample was compared to the sample at 40–60-cm depth of the same site. Enzyme names: *bssABC*, benzylsuccinate synthase; *bbsEF*, succinyl-CoA:(*R*)-4-isopropylbenzylsuccinate CoA-transferase; *bbsG*, (*R*)-benzylsuccinyl-CoA dehydrogenase; *bbsH*, phenylitaconyl-CoA hydratase; *bbsCD*, 2-[hydroxy(phenyl)methyl]succinyl-CoA dehydrogenase; *bbsAB*, benzoylsuccinyl-CoA thiolase; *ebdABC*, ethylbenzene dehydrogenase; *ped*, (*S*)-1-phenylethanol dehydrogenase; *apc12345*, acetophenone carboxylase; *bal.*, benzoylacetate-CoA ligase; *xccAC*, 4-hydroxyacetophenone carboxylase; *tioL*, predicted thiolase; *ppsABC*, phenylphosphate synthetase; *ppcABCD*, phenylphosphate carboxylase; *hcrAB/hbaBCD*, 4-hydroxybenzoyl-CoA reductase; *ald/aor6*, benzaldehyde dehydrogenase; *bclA/bzdA/hbaA*, 4-hydroxybenzoate-CoA/benzoate-CoA ligase; *bcrABCD/bamBC/badDEFG/bzdNOPQ*, benzoyl-CoA reductase; *dch/bamR*, cyclohex-1,5-diene-1-carbonyl-CoA hydratase; *had/bamQ*, 6-hydroxycyclohex-1-ene-1-carbonyl-CoA dehydrogenase; *oah/bamA*, 6-oxocyclohex-1-ene-1-carbonyl-CoA hydrolase; *masDEC/assABC*, (1-methylalkyl)succinate synthase; *assK*, AMP-dependent CoA ligase/synthetase; *citA/gltA*, citrate synthase; *acnAB*, aconitate hydratase; *icd/idh*, isocitrate dehydrogenase; *korAB*, 2-oxoglutarate:ferrodoxin oxidoreductase; *sucCD*, succinyl-CoA ligase; *frdABCD/sdhABCD*, fumarate reductase/succinate dehydrogenase; *fumABC*, fumarate hydratase; *mdh/mqo*, malate dehydrogenase/malate:quinone oxidoreductase; *fdhAB*, formate dehydrogenase; *fhs*, formate-tetrahydrofolate ligase; *folD/fchA*, methylenetetrahydrofolate dehydrogenase/methenyltetrahydrofolate cyclohydrolase; *metF*, methylenetetrahydrofolate reductase; *cooFS/coxSML*, carbon monoxide dehydrogenase; *acsCD/cdhABCDE*, carbon monoxide dehydrogenase/acetyl-CoA synthase; *pta*, phosphate acetyltransferase; *ackA*, acetate kinase

**Fig. 4:**
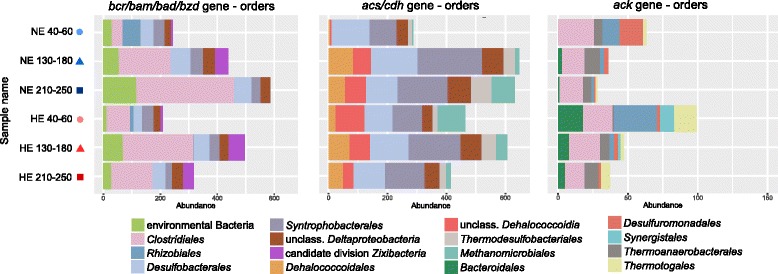
Taxonomic affiliation of metagenomic reads for representative genes. The seven most abundant orders are shown in each graph; each of the remaining orders represented less than 40, 69, and 9 reads in the samples, respectively. Enzyme names: *bcrABCD/bamBC/badDEFG/bzdNOPQ*, benzoyl-CoA reductase; *acsCD/cdhABCDE*, carbon monoxide dehydrogenase/acetyl-CoA synthase; *ackA*, acetate kinase

We further annotated our data using custom databases for all known genes coding for enzymes involved in the anaerobic degradation of hydrocarbons (addition to fumarate, hydroxylation, and assumed carboxylation) and could detect the presence of all genes in the analyzed metagenomes. Most were present at low abundance in all samples and showed no clear differences between the two sites. In contrast, the genes required for the anaerobic degradation of phenolic compounds to CO_2_ (*pps*, *ppc*, *hcr/hba*, *bcr/bam/bad/bzd*, *dch/bamR*, *had/bamQ* and *oah/bamA*, as well as key genes of the Wood-Ljungdahl pathway) showed a site-specific pattern. Whereas at the NE site, reads associated to these genes strongly increased with increasing depth, at the HE site a more even distribution was observed with a slight decrease at the bottom layer (Fig. 3). Taxonomic analysis of the key gene *bcr/bam/bad/bzd* (coding for benzoyl-CoA reductase) revealed that most reads were affiliated with *Clostridiales* (mainly *Peptococcaceae*) and these followed a similar site-specific pattern (Fig. 4). The latter were also identified as the main carriers of the *ack* gene (coding for acetate kinase) in all samples but they do not possess the gene *acs/cdh* (coding for carbon monoxide dehydrogenase/acetyl-CoA synthase).

### Comparison of Keri Lake metagenomes to those from other oil-impacted environments

To see if our findings can also be observed in other ecosystems with a long history of oil contamination, we compared our datasets to publicly available metagenomes from oil contaminated ecosystems available on MG-RAST [see Additional file 3: Table S2]. We categorized these into two groups: stabilized and perturbed. As stabilized we defined the ecosystems characterized by a long history of continuous and persistent exposure to oil, which has allowed the adaptation of the microbiomes to its presence. The dataset includes four metagenomes originating from one water sample from the Pitch Lake in La Brea (Trinidad and Tobago) [37] and three water samples from oil sand tailing ponds in Alberta (Canada) [58], all of which have been exposed to hydrocarbons for more than 30 years. As perturbed, we defined the ecosystems impacted by relatively recent anthropogenic discontinuous oil spills/leakages and which are presumed to harbor microbiomes “untrained”/non-adapted to oil. This dataset includes three metagenomes from marine sediments after the Deepwater Horizon oil spill (Gulf of Mexico) [30], three metagenomes from soils contaminated by leakages of polyaromatic hydrocarbons from a former oil refinery in Xaloztoc (Mexico) and one metagenome from soil contaminated by a BTEX (benzene, toluene, ethylbenzene, xylenes) leakage from an oil refinery in Cubatao (Brazil).

**Fig. 5:**
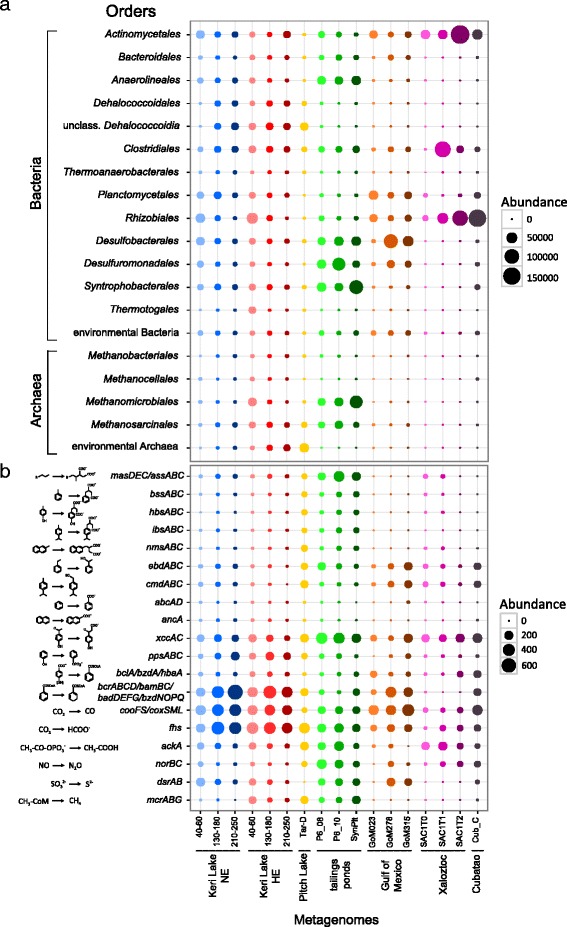
Comparison of Keri Lake metagenomes to publicly available datasets. **a** Abundances of selected prokaryotic orders in Keri Lake and publicly available metagenomes. **b** Absolute abundances of selected genes coding for enzymes involved in the pathways of anaerobic degradation of alkanes and aromatic compounds, Wood-Ljungdahl pathway, denitrification, sulfate reduction, and methanogenesis in Keri Lake and publicly available metagenomes

A taxonomic comparison of all samples revealed several similarities between the microbial communities at the HE site of Keri Lake and the other stabilized environments (Fig. 5a). High abundances of several anaerobic prokaryotic orders (*Anaerolineales*, unclassified *Dehalococcoidia, Methanomicrobiales*, and *Methanosarcinales*) were observed on these metagenomes. Archaeal sequences constituted a considerable part, particularly ~ 2.5% of the total number of reads compared to ~ 0.6% in the perturbed ecosystems, with most assigned to methanogens. However, differences in the abundance of sulfate reducers were visible. The orders *Desulfobacterales*, *Desulfuromonadales*, and *Syntrophobacterales* were present at low abundance at the HE site of Keri Lake, but were identified as key players in the samples from the oil sand tailing ponds as well as the deep sea sediments of the Gulf of Mexico.

**Fig. 6:**
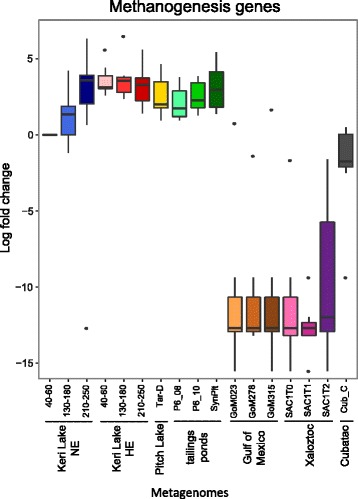
Compared abundances of the genes of methanogenesis in all studied metagenomes. Boxplots depict the log fold changes of the abundances of all genes coding for the enzymes of methanogenesis for each metagenome. All samples were compared to the NE 40–60-cm sample

An in-depth comparison of the functional potential of Keri Lake with the other metagenomes revealed small-scale differences in the abundances of genes representative for the anaerobic degradation of hydrocarbons among the different sites but no trend related to the history of contamination (Fig. 5b). The genes coding for alkyl−/arylalkylsuccinate synthases (*mas/ass*, *bss*, *hbs*, *ibs*, *nms*) had a higher abundance in the metagenomes of the Pitch Lake and the oil sand tailing ponds compared to those from Keri Lake. The genes for anaerobic hydroxylation reactions of aromatic hydrocarbons (*ebd* and *cmd*) were present in all metagenomes, whereas genes for putative carboxylases (*abc* and *anc*) were not detected in any of the analyzed environments. The gene *bcr/bam/bad/bzd* as well as two genes of the Wood-Ljungdahl pathway (*coo* and *fhs*) showed a higher abundance in the samples from Keri Lake and the Gulf of Mexico, compared to the other metagenomes. In contrast, the gene encoding acetate kinase (*ack*) had a lower abundance in these environments. Among the anaerobic respiratory pathways, the *mcr* gene had a > 2 times higher abundance compared to *nor* and *dsr* in Keri Lake and Pitch Lake samples. The oil sand tailing ponds represented the only environment with similar abundances of genes of the different respiratory pathways (Fig. 5b). We further determined the log fold changes in normalized reads for all genes involved in methanogenesis between each sample and the NE site (40–60-cm depth). Figure 6 summarizes these changes in box plots and reveals a higher abundance of genes involved in methanogenesis in the stabilized compared to all perturbed environments.

## Discussion

### Hydrocarbon composition and availability of electron acceptors

The prevalence of long-chain *n*-alkanes with odd numbers of carbon atoms at the NE site most likely derives from waxes of higher plants and thus indicates a terrestrial organic matter input. Additionally, the high relative abundance of *n*-tetracosane (C_24_) can be attributed to an origin from reeds of the genus *Phragmites* [59]. At the HE site, this characteristic biogenic hydrocarbon signature was completely overlaid by that of the asphalt oil [see Additional file 1: Figure S1B]. The detected hopane biomarkers are formed from triterpenoid precursors of the hopanoid type during oil generation, which predominantly originate from bacterial biomass. In pristine crude oils, these compounds typically occur as trace constituents. As they are oil constituents resistant to biodegradation, their enrichment in the asphalt oil of the HE site together with the observed unresolved complex hydrocarbon mixture is a clear indication for heavy microbial alteration of the original oil composition [60, 61].

We further observed a complete depletion of sulfate at the HE site [see Additional file 1: Figure S1A] that can reasonably be attributed to the degradation of the abundant petroleum constituents, which is in accordance with our hypothesis that microbial degradation of the oil depletes electron acceptors. The high concentrations of nitrate at the same site, compared to the NE site, were unexpected though. Nitrate respiration provides more energy to microbes compared to sulfate reduction or methanogenesis, thus the use of nitrate should be preferred for the degradation of hydrocarbons. This does not seem to be the case in the studied ecosystem. It is possible that the presence of heavy asphalt oil in the HE sediment may inhibit the biological processes that remove nitrate, e.g., denitrification, or the available constituents of the asphalt oil cannot be processed by denitrifying bacteria.

### Impact of asphalt oil on microbial community structure and function

In this study, the comparison of metagenomes from sediment samples obtained from different depths of the asphalt oil-exposed HE site with the non-exposed NE site at Keri Lake allowed us to detect changes in the structure and the functional potential of the indigenous microbiomes. Any difference between these sites can be regarded as the direct or indirect result of the presence of asphalt oil, since both sampling sites are located in the same geographical area and are exposed to the same seasonal climate conditions and water dynamics. The number of samples we could obtain was limited due to the strict protective regulations that apply to the area; Keri Lake is part of Laganas Bay which is a Natura 2000 site, thus any human activities are minimized. More sampling points would be necessary to statistically confirm the level of significance of the observed differences.

Considering the significant input of terrestrial organic matter, we assume the presence of lignin-derived phenolic compounds in particular in the bottom layer of the NE site. Accordingly, with increasing depth, an increase in the potential for the degradation of these phenolic compounds was observed (Fig. 3). The strong oil impact apparently altered this depth-related pattern at the HE site. Here, plant-derived material was scarce and the available organic material was composed mainly of higher molecular weight oil constituents throughout the sedimentary column. The abundance of the genes involved in the degradation of phenolic compounds was high in all depths and displayed no maximum at the bottom layer, while the rest of the genes involved in the anaerobic degradation of hydrocarbons had rather low abundances. The observed pattern can also be attributed to the depletion of the asphalt oil in low molecular weight hydrocarbons due to biodegradation, which might have already started after the accumulation of the oil in its subsurface reservoir [62]. The remaining high molecular weight constituents of the asphalt oil typically show very low water solubility and adsorb easily to sediment particles, which reduces their bioavailability. Under these conditions, microorganisms may prefer to utilize other less recalcitrant substrates available in the sediment. Alternatively, there might be presently unknown genes/enzymes and undetectable pathways involved in the degradation of high molecular weight hydrocarbons, thus a significant part of the degradation potential of the communities may have been hidden in the reads that could not be assigned to functions.

The increased potential for phenol degradation with depth at the NE site was paralleled by a transition from denitrification/sulfate reduction to methanogenesis. One could speculate about a switch in the respiratory pathway coupled to the degradation of lignin compounds towards methanogenesis in the deeper layers of Keri Lake sediments. At the HE site, however, the genes for methanogenesis expanded from the bottom to the top layer (Fig. 2). The simultaneous decrease in the potential for the other respiratory pathways compared to the NE site shows that methanogenesis is not only favored but also predominates over denitrification and sulfate reduction and that the respiratory profile of the HE site is clearly influenced by the presence of asphalt oil.

The increased potential for methanogenesis was also reflected by the taxonomic profiles of our samples where the relative abundance of *Euryarchaeota* increased in the samples from the HE site compared to those from the NE site (Fig. 1). Previous studies have also shown that *Euryarchaeota* increase in anoxic environments after accidental spills, but other known degraders of aromatic hydrocarbons and *n*-alkanes have been observed to quickly increase in abundance as well. Mason et al. [31] and Kimes et al. [30] suggested that the Deepwater Horizon accident mainly enriched denitrifying *Gamma-* and sulfate-reducing *Deltaproteobacteria* respectively, as key hydrocarbon degraders in the impacted marine sediments. Acosta-González et al. [25] though observed a decrease in *Deltaproteobacteria* 3 years after the contamination of marine sediments by the Prestige oil spill, which is in line with our data. These may suggest that syntrophic methanogenic consortia predominate over early responding *Proteobacteria* when the microbiomes remain exposed to oil for longer time periods.

The process of methanogenesis is favored when inorganic electron acceptors are depleted or their bioavailability is decreased in hydrocarbon-rich environments. Oil reservoirs are often free of sulfate, similar to the HE site. Methanogenesis is commonly associated with the biodegradation of crude oil in reservoirs [63] and is likely the main mechanism responsible for the formation of heavy oil in these ecosystems [64]. Methanogenic classes of the phylum *Euryarchaeota* have been repeatedly observed in oil reservoirs worldwide and considered part of their core microbiome [65].

The taxonomic profiles of the samples from the HE site showed also other similarities to methanogenic hydrocarbon-degrading enrichment cultures originating from oil reservoir production waters, oil sand tailings or contaminated anoxic sediments. *Clostridia* members, including *Peptococcaceae*, have repeatedly been detected in methanogenic cultures amended with aromatic hydrocarbons or oil mixtures [66–71] as primary fermenters of the hydrocarbons in syntrophic interactions with methanogenic *Euryarchaeota*. The detection of the *ack* gene from *Clostridiales* in our samples suggests that they have the potential to generate and provide acetate to other taxa, e.g., *Thermotogales* and/or *Methyloceanibacter*. Members of *Thermotogales* have several times been detected in high-temperature oil reservoirs [65] and methanogenic enrichment cultures [66, 67, 70, 72] and considered to be secondary fermenters during hydrocarbon degradation. Combined with the detection of several different methanogens in our samples, these suggest complex syntrophic interactions in Keri Lake sediments, probably involving more than two partners.

### Long history of oil exposure affects the structure and function of microbiomes

Based on the taxonomic and functional potential revealed by the metagenomes (Fig. 5), the HE samples from Keri Lake were similar to the other stabilized ecosystems, especially from the Pitch Lake, used for comparison in the framework of this study. Microbiomes of long-term oil-exposed habitats are depleted in electron acceptors. Increased numbers of *Euryarchaeota* and high methanogenic potential were common for these environments (Fig. 6), underpinning the prominent role of syntrophic methanogenic interactions in these ecosystems. Only the metagenomes from the oil sand tailing ponds showed a high abundance of genes linked to denitrification and sulfate reduction as well. This is possibly explained by the high dynamics in these habitats with frequent water recycling and external input of new contaminated material [73], including electron donors and acceptors. However, as seen from the data in our study, the input of electron acceptors like nitrate might not shift the redox processes in every case. Denitrifiers are possibly blocked under specific environmental settings, e.g., the presence of heavy asphalt oil.

The order of *Clostridiales* was detected in high abundance in all studied sites despite their difference in the history of oil exposure. This finding supports their general ability of *Clostridiales* members to utilize hydrocarbons under various conditions [68, 74–77] and tolerate the exposure to oil. The abundances of gene coding for enzymes of the anaerobic degradation of hydrocarbons however were dependent on the study site (Fig. 5b). These most likely reflect the differences in oil quality in terms of composition of hydrocarbons. Thus, we consider that the hydrocarbonoclastic potential of the microbiomes in each environment is influenced by the chemical composition of the oil, which can be very dissimilar among different study sites. It is possible though that we were not able to identify clear trends as a result of the introduced bias due to differences in the handling of the different metagenomes (e.g., DNA extraction, library preparation, and sequencing method). We expect that the change in the oil composition due to historic weathering affects the abundances of genes associated with the degradation of hydrocarbons in ecosystems exposed to petroleum for long time periods, but this has yet to be tested.

## Conclusions

The analysis of microbiomes in a surface land-to-sea transition ecosystem with a natural occurrence of asphalt oil and the comparison with other datasets suggest that more than 2500 years of exposure to oil shapes microbiomes with the potential for hydrocarbon degradation linked to methanogenesis, thus favors syntrophic methanogenic interactions. In-depth sequencing of our metagenomes would have allowed the assembly of microbial genomes, the accurate identification of metabolic pathways and their connection to the extracted genomes. Since the present study was based on DNA sequencing, it provides no information about the expression of the detected genes. Further metatranscriptomic/metaproteomic/metametabolomic analyses may offer more insights into the processes which are actually active in situ. Additionally, lab experiments under well-defined conditions are needed to directly connect our findings to the depletion of electron acceptors (e.g., sulfate) in the oil-exposed sediment.

## Additional files

Additional file 1: Figure S1. Geographical overview of the study sites. (A) Map of Greece showing the Hellenic trench, the plate boundaries and the major fault systems modified after Panagiotaras et al. [79]. (B) Geological map of Zakynthos Island modified after the Institute of Geology and Mineral Exploration of Greece (1980) and Avramidis et al. [80]. (C) Aerial view of Keri Lake with the location of the NE and HE sites. (PDF 2443 kb)

Additional file 2: Table S1. Selected genes quantified in Keri Lake metagenomes. The gene subunits and the corresponding Uniprot entries of the protein sequences included in each database are listed. (XLS 47 kb)

Additional file 3: Table S2. MG-RAST metagenomes analyzed and compared to Keri Lake samples and their features. The metagenomes are grouped in stabilized and perturbed based on the time they are exposed to oil. (XLS 35 kb)

Additional file 4: Figure S2. Geochemical characterization of the study sites. (A) Concentrations of nitrate and sulfate in mg g^−1^ dry sediment. Values represent the average of two independent samples analyzed. (B) Partial gas chromatograms of the saturated hydrocarbon fractions of samples from the NE (upper panel) and HE (lower panel) sites. Green dots, *n*-alkanes; orange dots, hopanes; IS, internal standard (*α*-androstane); EOM, extractable organic matter. (PDF 112 kb)

Additional file 5: Table S3. Summary of samples obtained from the NE and HΕ sites to asphalt oil. (XLS 36 kb)

Additional file 6: Figure S3. Rarefaction curves of the taxonomic analysis in Keri Lake metagenomic samples at the (A) phylum and (B) order level. The deep sequenced samples are presented after normalization by subsampling to ~2.12 million reads. (PDF 72 kb)

Additional file 7: Figure S4. Distribution and abundance of prokaryotic taxa at different depths of the NE and HE sites. The ten most abundant phyla are depicted as distinct colors. Individual data points represent different orders; the size indicates the order’s abundance while the position on the plot shows the proportion in the three depths. (PDF 42 kb)

Additional file 8: Figure S5. Rarefaction curves of the functional analysis in Keri Lake metagenomic samples (A) for the detected KEGG Orthology numbers of the 10 most abundant phyla and (B) for the 68 genes of interest. The deep sequenced samples are presented after normalization by subsampling to ~2.12 million reads. (PDF 46 kb)

## Abbreviations

HE: Site highly exposed to oil
NE: Site non-exposed to oil
