# Correction to: More than 2500 years of oil exposure shape sediment microbiomes with the potential for syntrophic degradation of hydrocarbons linked to methanogenesis

**DOI:** 10.1186/s40168-017-0354-7

**Published:** 2017-10-11

**Authors:** Antonios Michas, Gisle Vestergaard, Kathleen Trautwein, Pavlos Avramidis, Dimitris G. Hatzinikolaou, Constantinos E. Vorgias, Heinz Wilkes, Ralf Rabus, Michael Schloter, Anne Schöler

**Affiliations:** 1 Research Unit Comparative Microbiome Analysis (COMI), Helmholtz Zentrum München, Ingolstaedter Landstraße 1, D-85764 Neuherberg, Germany; 2 General and Molecular Microbiology, Institute for Chemistry and Biology ofthe Marine Environment (ICBM), Carl von Ossietzky University Oldenburg, Carl-von-Ossietzky-Straße 9-11, 26111 Oldenburg, Germany; 3 Department of Geology, University of Patras, Panepistimioupoli Patron, 26504 Rio-Patras, Greece; 4 Department of Biology, National and Kapodistrian University of Athens, Zografou University Campus, 15784 Athens, Greece; 5 Organic Geochemistry, Institute for Chemistry and Biology of the Marine Environment (ICBM), Carl von Ossietzky University Oldenburg, Carl-von-Ossietzky-Straße 9-11, 26129 Oldenburg, Germany

## Correction

Following publication of the original article [1], the authors requested two minor changes. The first is to the name of one of the genes of the TCA cycle (*fumABC*) in Fig. 3. The correct figure with the name of the gene added is included below.

**Fig. 3:**
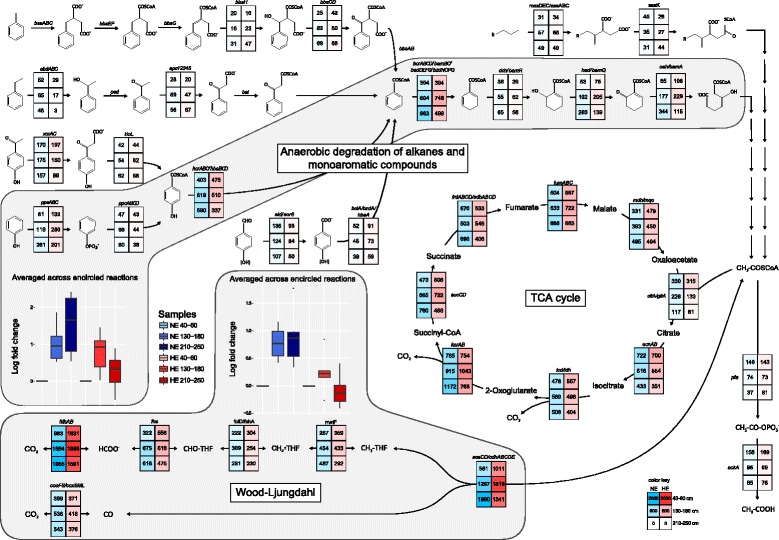
Reconstruction of the complete degradation of *n*-alkanes and monoaromatic compounds to CO_2_. The normalized absolute abundances of the genes for each step are given in the respective cells. The selection of the genes for the anaerobic degradation of aromatic compounds was based on the proteogenomics-based reconstruction of their catabolism in the denitrifying *Aromatoleum aromaticum* EbN1 [78] and the sulfate-reducing *Desulfobacula toluolica* Tol2 [15]. Only genes with abundances higher than 30 reads in at least one sample are presented. The boxplots depict the log fold changes of the abundances of all genes coding for the enzymes of the anaerobic degradation of phenolic compounds and the Wood-Ljungdahl pathway, respectively. Each sample was compared to the sample at 40–60-cm depth of the same site. Enzyme names: *bssABC*, benzylsuccinate synthase; *bbsEF*, succinyl-CoA:(*R*)-4-isopropylbenzylsuccinate CoA-transferase; *bbsG*, (*R*)-benzylsuccinyl-CoA dehydrogenase; *bbsH*, phenylitaconyl-CoA hydratase; *bbsCD*, 2-[hydroxy(phenyl)methyl]succinyl-CoA dehydrogenase; *bbsAB*, benzoylsuccinyl-CoA thiolase; *ebdABC*, ethylbenzene dehydrogenase; *ped*, (*S*)-1-phenylethanol dehydrogenase; *apc12345*, acetophenone carboxylase; *bal.*, benzoylacetate-CoA ligase; *xccAC*, 4-hydroxyacetophenone carboxylase; *tioL*, predicted thiolase; *ppsABC*, phenylphosphate synthetase; *ppcABCD*, phenylphosphate carboxylase; *hcrAB/hbaBCD*, 4-hydroxybenzoyl-CoA reductase; *ald/aor6*, benzaldehyde dehydrogenase; *bclA/bzdA/hbaA*, 4-hydroxybenzoate-CoA/benzoate-CoA ligase; *bcrABCD/bamBC/badDEFG/bzdNOPQ*, benzoyl-CoA reductase; *dch/bamR*, cyclohex-1,5-diene-1-carbonyl-CoA hydratase; *had/bamQ*, 6-hydroxycyclohex-1-ene-1-carbonyl-CoA dehydrogenase; *oah/bamA*, 6-oxocyclohex-1-ene-1-carbonyl-CoA hydrolase; *masDEC/assABC*, (1-methylalkyl)succinate synthase; *assK*, AMP-dependent CoA ligase/synthetase; *citA/gltA*, citrate synthase; *acnAB*, aconitate hydratase; *icd/idh*, isocitrate dehydrogenase; *korAB*, 2-oxoglutarate:ferrodoxin oxidoreductase; *sucCD*, succinyl-CoA ligase; *frdABCD/sdhABCD*, fumarate reductase/succinate dehydrogenase; *fumABC*, fumarate hydratase; *mdh/mqo*, malate dehydrogenase/malate:quinone oxidoreductase; *fdhAB*, formate dehydrogenase; *fhs*, formate-tetrahydrofolate ligase; *folD/fchA*, methylenetetrahydrofolate dehydrogenase/methenyltetrahydrofolate cyclohydrolase; *metF*, methylenetetrahydrofolate reductase; *cooFS/coxSML*, carbon monoxide dehydrogenase; *acsCD/cdhABCDE*, carbon monoxide dehydrogenase/acetyl-CoA synthase; *pta*, phosphate acetyltransferase; *ackA*, acetate kinase

The second is to a sentence in the Authors’ Contribution section which should be changed from:

*KT, RR, DGH, and MS designed the study. AM, KT, PA, DGH, and CEV collected the samples from Keri Lake. HW analyzed the hydrocarbon content and composition. AM, GV, and AS generated and analyzed the sequence data. AM, RR, MS, and AS conceptualized and wrote the manuscript. All authors contributed to revisions and approved the final manuscript.*

To:

*KT, RR, DGH, and MS designed the study. PA designed the geological study. AM, KT, PA, DGH, and CEV collected the samples from Keri Lake. HW analyzed the hydrocarbon content and composition. AM, GV, and AS generated and analyzed the sequence data. AM, RR, MS, and AS conceptualized and wrote the manuscript. All authors contributed to revisions and approved the final manuscript.*

The original article has been corrected.
