# Supplementary figures and images for: More than 2500 years of oil exposure shape sediment microbiomes with the potential for syntrophic degradation of hydrocarbons linked to methanogenesis
Source: Microbiome. 2017 Sep 11;5:118. doi: 10.1186/s40168-017-0337-8 (PMC5594585; doi:10.1186/s40168-017-0337-8)

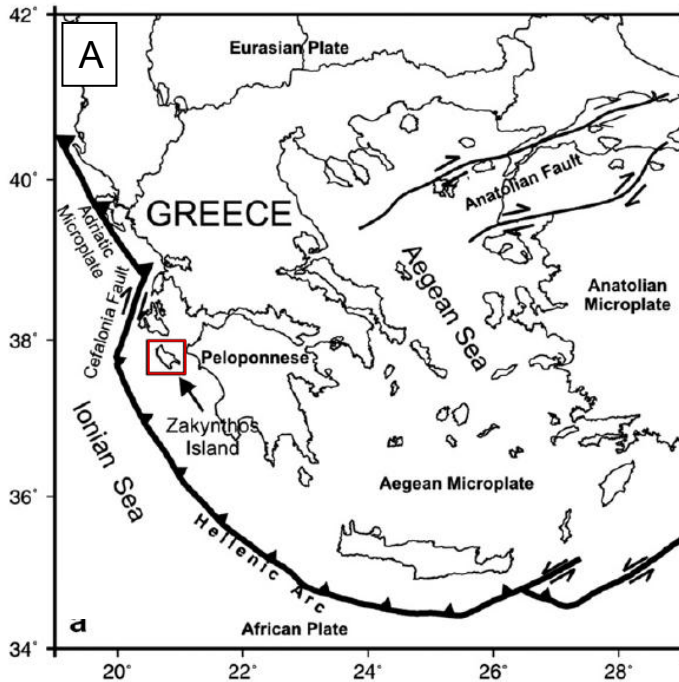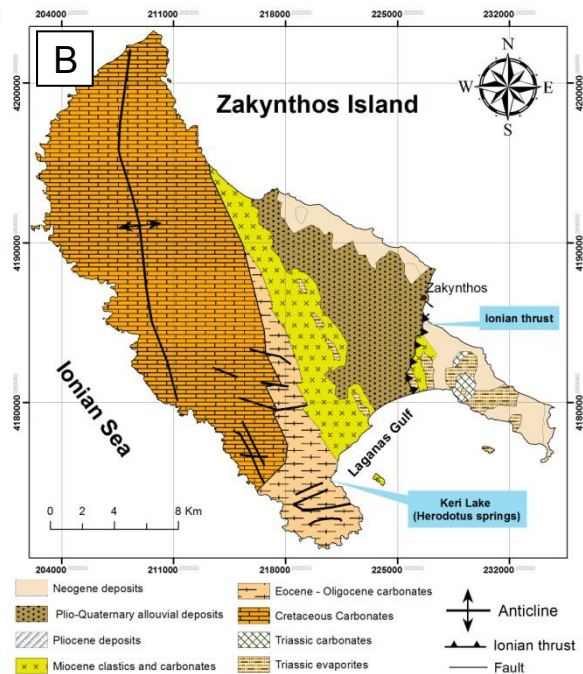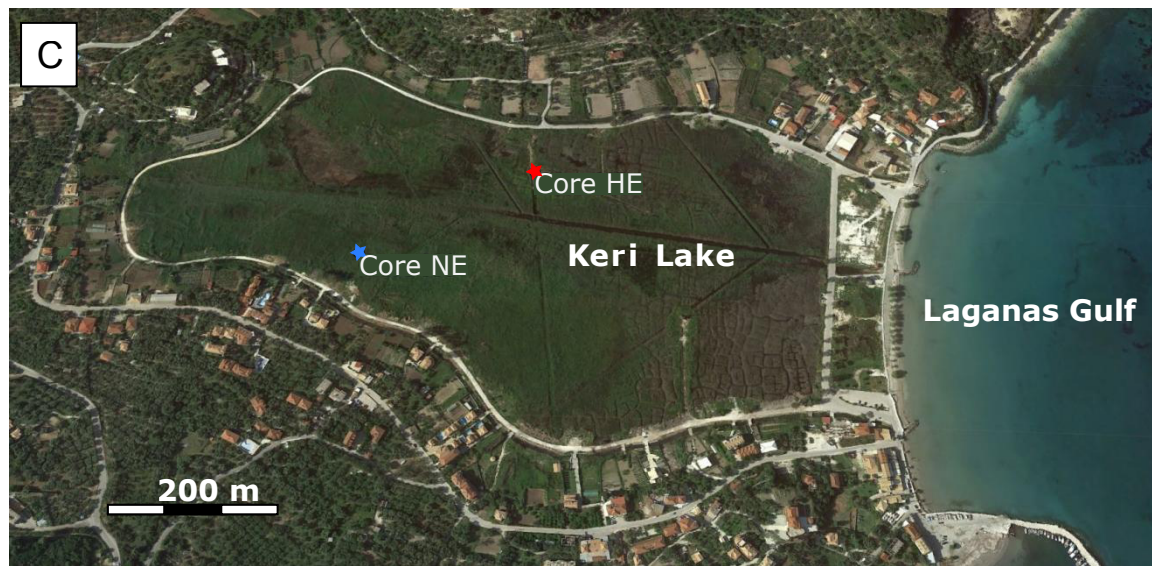

Supplement: Supplementary file 1 — Geographical overview of the study sites. (A) Map of Greece showing the Hellenic trench, the plate boundaries and the major fault systems modified after Panagiotaras et al. [79]. (B) Geological map of Zakynthos Island modified after the Institute of Geology and Mineral Exploration of Greece (1980) and Avramidis et al. [80]. (C) Aerial view of Keri Lake with the location of the NE and HE sites. (PDF 2443 kb) [file 40168_2017_337_MOESM1_ESM.pdf]

(A)

Nitrate concentration ( $\text{mg g}^{-1}$ )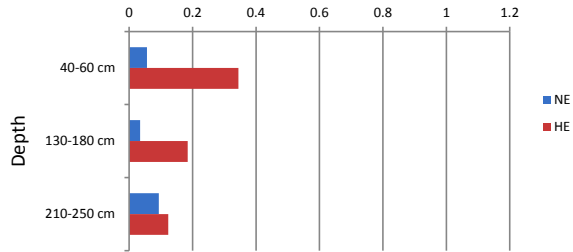Sulfate concentration ( $\text{mg g}^{-1}$ )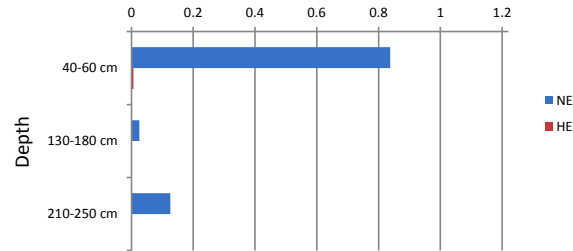

(B)

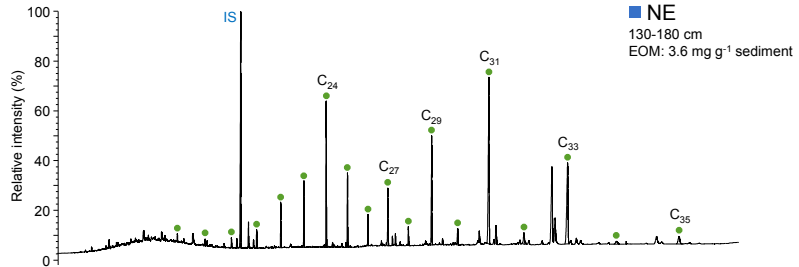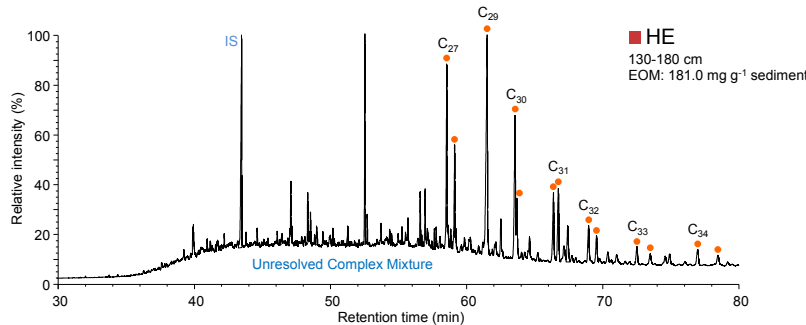

Supplement: Supplementary file 4 — Geochemical characterization of the study sites. (A) Concentrations of nitrate and sulfate in mg g−1 dry sediment. Values represent the average of two independent samples analyzed. (B) Partial gas chromatograms of the saturated hydrocarbon fractions of samples from the NE (upper panel) and HE (lower panel) sites. Green dots, n-alkanes; orange dots, hopanes; IS, internal standard (α-androstane); EOM, extractable organic matter. (PDF 112 kb) [file 40168_2017_337_MOESM4_ESM.pdf]

(A)

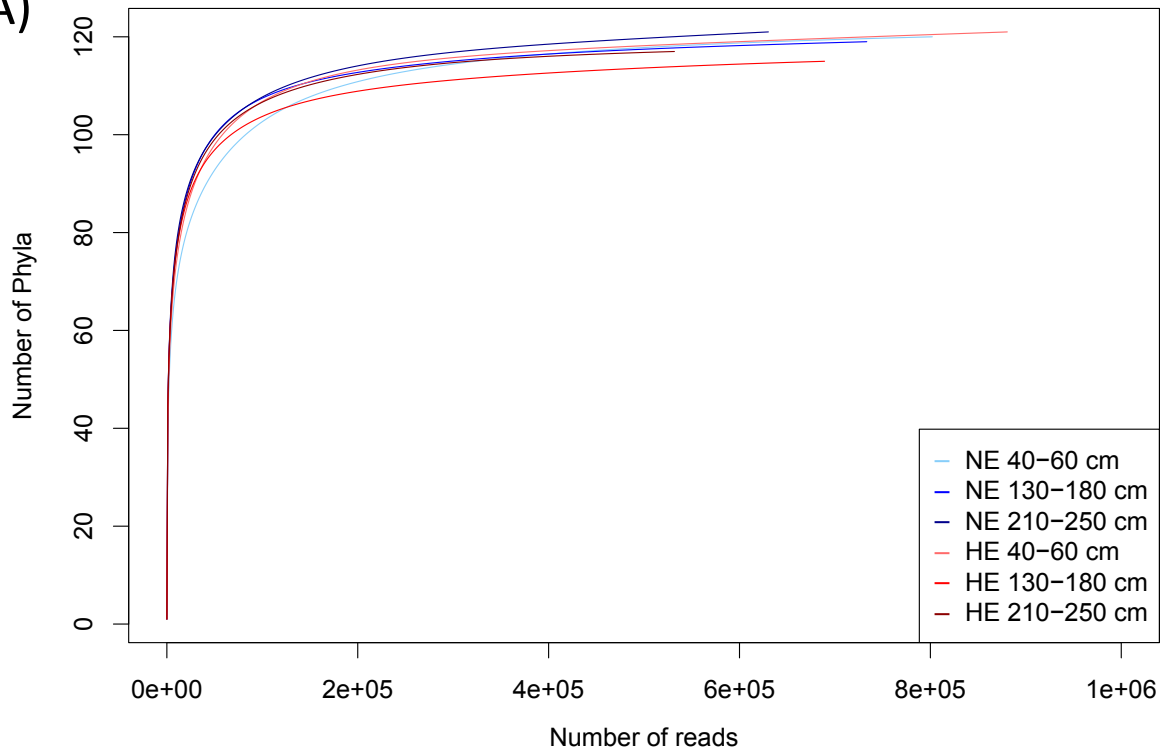

(B)

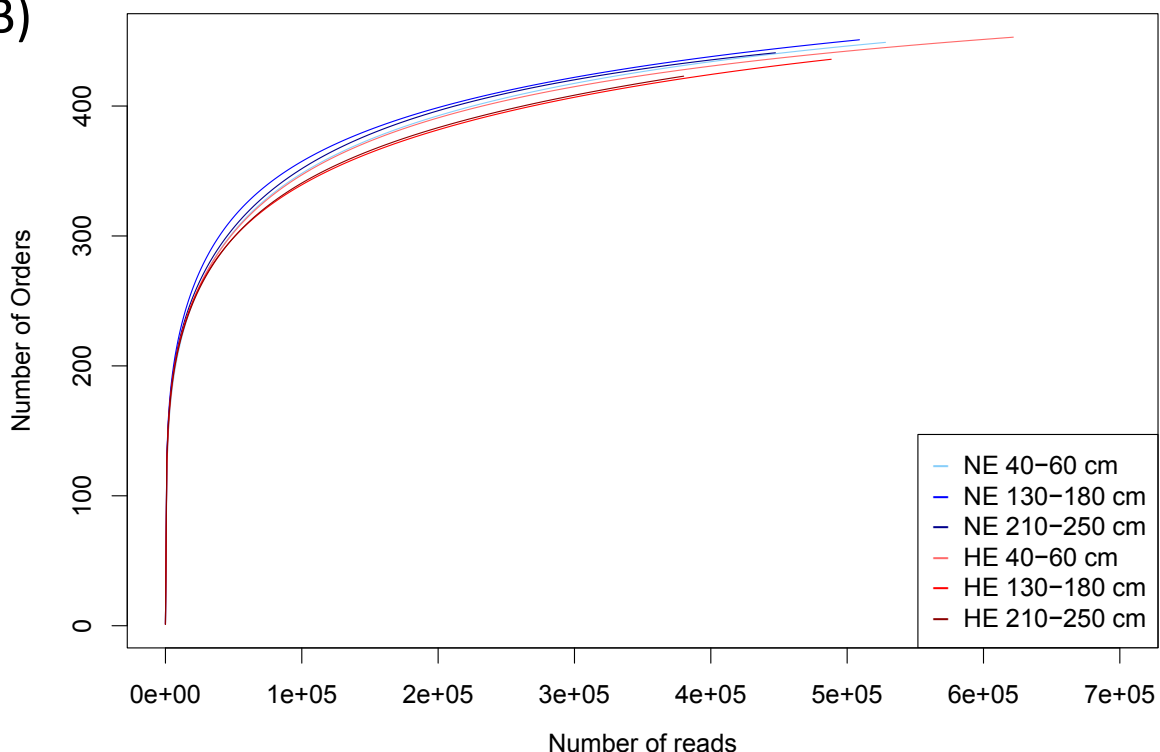

Supplement: Supplementary file 6 — Rarefaction curves of the taxonomic analysis in Keri Lake metagenomic samples at the (A) phylum and (B) order level. The deep sequenced samples are presented after normalization by subsampling to ~2.12 million reads. (PDF 72 kb) [file 40168_2017_337_MOESM6_ESM.pdf]

(A)

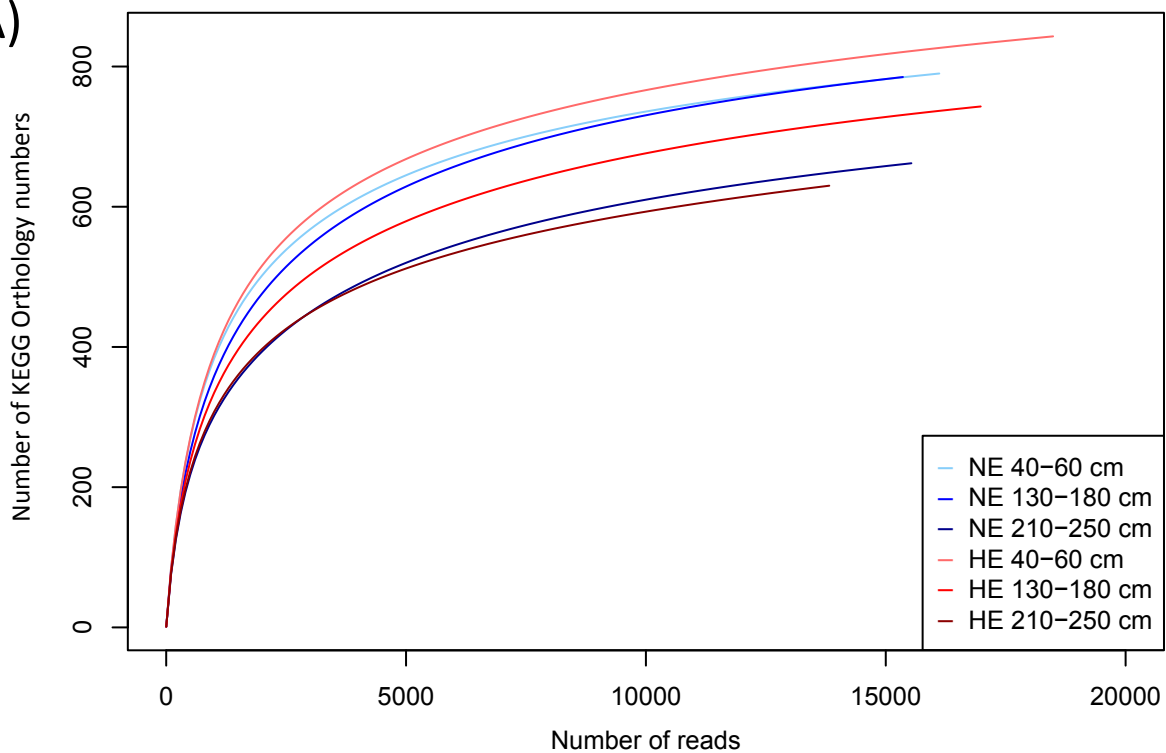

(B)

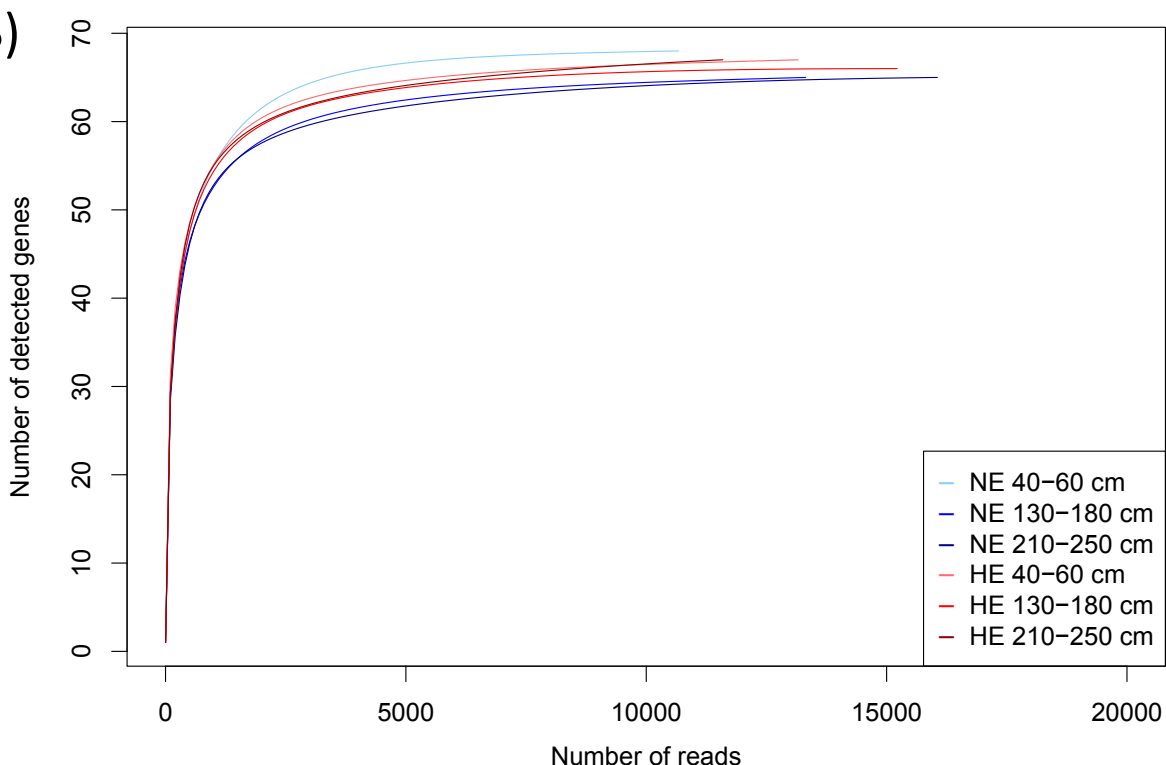

Supplement: Supplementary file 7 — Distribution and abundance of prokaryotic taxa at different depths of the NE and HE sites. The ten most abundant phyla are depicted as distinct colors. Individual data points represent different orders; the size indicates the order’s abundance while the position on the plot shows the proportion in the three depths. (PDF 42 kb) [file 40168_2017_337_MOESM7_ESM.pdf]

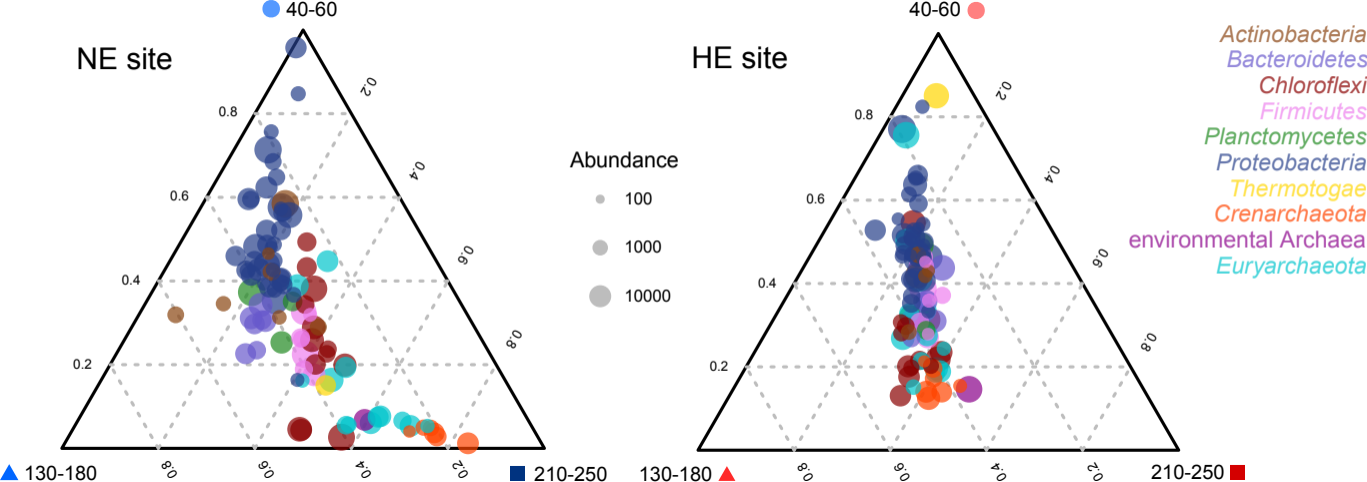

Supplement: Supplementary file 8 — Rarefaction curves of the functional analysis in Keri Lake metagenomic samples (A) for the detected KEGG Orthology numbers of the 10 most abundant phyla and (B) for the 68 genes of interest. The deep sequenced samples are presented after normalization by subsampling to ~2.12 million reads. (PDF 46 kb) [file 40168_2017_337_MOESM8_ESM.pdf]
